# Monoclonal Antibodies for Protozoan Infections: A Future Reality or a Utopic Idea?

**DOI:** 10.3389/fmed.2021.745665

**Published:** 2021-10-12

**Authors:** Silvia Stefania Longoni, Natalia Tiberti, Zeno Bisoffi, Chiara Piubelli

**Affiliations:** ^1^Department of Infectious-Tropical Diseases and Microbiology, Istituto di Ricovero e Cura a Carattere Scientifico (IRCCS), Sacro Cuore Don Calabria Hospital, Verona, Italy; ^2^Department of Diagnostics and Public Health, University of Verona, Verona, Italy

**Keywords:** monoclonal antibody, toxoplasmosis, Chagas disease, malaria, leishmaniasis, protozoa

## Abstract

Following the SARS-CoV-2 pandemic, several clinical trials have been approved for the investigation of the possible use of mAbs, supporting the potential of this technology as a therapeutic approach for infectious diseases. The first monoclonal antibody (mAb), Muromonab CD3, was introduced for the prevention of kidney transplant rejection more than 30 years ago; since then more than 100 mAbs have been approved for therapeutic purposes. Nonetheless, only four mAbs are currently employed for infectious diseases: Palivizumab, for the prevention of respiratory syncytial virus (RSV) infections, Raxibacumab and Obiltoxaximab, for the prophylaxis and treatment against anthrax toxin and Bezlotoxumab, for the prevention of *Clostridium difficile* recurrence. Protozoan infections are often neglected diseases for which effective and safe chemotherapies are generally missing. In this context, drug resistance and drug toxicity are two crucial problems. The recent advances in bioinformatics, parasite genomics, and biochemistry methodologies are contributing to better understand parasite biology, which is essential to guide the development of new therapies. In this review, we present the efforts that are being made in the evaluation of mAbs for the prevention or treatment of leishmaniasis, Chagas disease, malaria, and toxoplasmosis. Particular emphasis will be placed on the potential strengths and weaknesses of biological treatments in the control of these protozoan diseases that are still affecting hundreds of thousands of people worldwide.

## Introduction

The *in vitro* production of murine monoclonal antibodies (mAbs) was first described in 1975 by Kohler and Milstein, a discovery that earned them the Nobel Prize in 1985 and that revolutionized the clinical practice and biomedical research (1–3). Since then, mAbs have been engineered and stable cell lines able to secrete specific immunoglobulins against the target antigen of interest have been obtained (4). Nowadays there are more than 100 mAbs approved by the US Food and Drug Administration (FDA) (5, 6) and/or by the European Medicines Agency (EMA) (7), and they are classified into four types: murine (–omab), chimeric (–ximab), humanized (~95% human, –zumab), and human (–umab) (3), with the latter being the most successful in terms of tolerability and efficacy. Most of the approved mAbs are used in the field of oncology and immunology, while only a few are directed against infectious diseases, in particular against the respiratory syncytial virus (RSV) (Palivizumab), the anthrax toxin (Raxibacumab and Obiltoxaximab) and the bacterium *Clostridium difficile* (Bezlotoxumab), for which they are used either for prophylaxis or treatment (6, 7). A therapy using mAbs against protozoan infections is completely missing.

Eleven out of the 20 priority neglected tropical diseases (NTDs) included in the World Health Organization (WHO) portfolio are parasitosis (8). The drugs currently employed to treat these diseases are at least 50 years old, present several side effects and are not 100% efficient partly due to recurrent drug resistance (9–15).

The lack of mAb therapies for parasitosis is to a certain extent due to the neglected status of these diseases, lashing mainly low resource countries, and to high commercial costs of this technology.

In the context of protozoan diseases, two strategies can be followed for the development and use of mAbs. The first consists in the use of antibodies that target host antigens, mostly immune factors. Such a strategy allows modulating host immunity to achieve a more effective response for parasite elimination or at limiting damages due to hyper-inflammation. The main advantages of this type of approach are (i) the possibility of exploiting drug repurposing, thus using drugs already developed, tested in clinical trials, and approved; (ii) the therapeutic efficacy is not undermined by the development of resistance or by antigenic variability; (iii) they might be found particularly useful during chronic infections in which the host response contributes to the pathology. Nonetheless, this strategy requires an in-depth knowledge of the mechanisms of host-pathogen interaction and of immunomodulation, which in the vast majority of the cases are far from being deciphered.

Alternatively, mAbs targeting directly parasitic antigens can be employed to induce parasite elimination through different mechanisms including antibody-dependent cellular cytotoxicity, antibody-dependent cellular phagocytosis, and complement-dependent cytotoxicity (16). The identification of the appropriate highly conserved targets for the development of such mAbs can however be cumbersome due to both the phenomenon of antigenic variation that characterizes most protozoa and variability between strains. Moreover, this strategy depends upon a wide knowledge of the parasite life cycle, biochemical processes, and adaptation mechanisms, which unfortunately is often limited.

With this review we intend to do revisit the state-of-the-art of mAb research for protozoan infections, summarizing the most relevant candidate therapeutics proposed and the different strategies. We will present how far research on this field has progressed, from *in vitro* and animal studies to clinical trials, and which are the main obstacles that have been encountered. In particular, we will deal with mAbs for leishmaniasis, Chagas disease, malaria, and toxoplasmosis, for which important experimental studies or clinical trials are ongoing, as summarized in Tables 1, 2. Possible strategies to overcome the current limits of this technology for the control of parasitic diseases in the context of human public health will also be discussed.

**Table 1:** mAb evaluated in *in vitro* and animal models for protozoan diseases treatment.

**mAb**	**Target**	**Protozoan disease**	**Model**	**Main effects**	**Reference**
Anti-PD-1	Host	Leishmaniasis	*in vitro*. Dog mononuclear cells	Reduction of parasite burden Increased release of NO, IL-4, and TNF	(17)
			*in vitro*. Dog phagocytes	Elimination of intracellular parasites	(18)
Anti-CD2	Host	Leishmaniasis	*in vivo*. Combined with conventional antimonial chemotherapy in BALB/c mice infected with *L. donovani*	Parasite elimination and parasite replication control	(19)
Anti-JAM-C	Host	Leishmaniasis	*in vivo*, C57BL/6 and BALB/C mice	Increased Th1 response leading to the reduction of skin lesions and parasite burden in resistant C57BL/6 mice; Boosted Th2 response promoting infection in susceptible BALB/c mice	(20)
Anti-TGFb	Host	Leishmaniasis	*in vivo*, CB6F1 mice	Rapid lesion healing and reduction in the number of parasites present in the skin lesion	(21)
Anti-CD25 mAb (PC61 hybridoma)	Host	Chagas disease	*in vivo*, experimentally infected BALB/c mice	Increase in IFN-γ and TNF-α production in CD acute stage	(22)
Anti-CD25 mAb (7D4 hybridoma)	Host	Chagas disease	*in vivo*, experimentally infected BALB/c mice	Reduced parasitemia and increased effector memory T cells and IFN-γ-TNF-α secreting cells during acute stage	(23)
Anti-CSP	Circumsporozoite protein	*P. falciparum*—sporozoite	*in vitro*: *Plasmodium falciparum* (NF54) *in vivo*: C57BL/6 mice infected with transgenic *P. berghei* sporozoites	Prevention of hepatocyte invasion	(24, 25)
Anti-PfRH5	Merozoite reticulocyte-binding protein homolog 5	*P. falciparum*—merozoite	*in vitro*: vaccine-induced anti-RH5 serum antibody (healthy, malaria-naive male subjects, and non-pregnant females). Six laboratory-adapted *P. falciparum* strains and clinical isolates.	Inhibition of merozoite invasion of erythrocytes	(26, 27)
			*in vitro*: isolated PBMC from vaccinated humans and HEK293T cells		
Anti-CyRPA	Cysteine-rich protective antigen	*P. falciparum*—merozoite	*in vitro*: *Plasmodium falciparum* (3D7)	Inhibition of merozoite invasion of erythrocytes	(28)
PfEBA175	EBA175	*P. falciparum*—merozoite	*in vitro*: *Plasmodium falciparum* (strains: Dd2, MCamp, 7G8, FCR3, HB3, DIV30, K39, KMVII, M190, and 3D7)	Inhibition of merozoite invasion of erythrocytes	(29, 30)
			*in vitro*: HEK-293T cells line		
PfAMA1	Apical membrane antigen 1	*P. falciparum*—merozoite	*in vitro*: *P. falciparum* (strains: 3D7A, HB3, and FCR3)	Inhibition of merozoite invasion of erythrocytes	(31)
MSP	Merozoite surface protein	*P. falciparum*—merozoite	*in vitro*: *Plasmodium falciparum* (3D7).	Inhibition of merozoite invasion of erythrocytes	(32, 33)
			*in vitro*: *Plasmodium knowlesi* (A1-H.1)		
Pfs48/45	Gametocyte surface protein	*P. falciparum*—gametocyte	*in vivo*: *Anopheles stephensi* mosquitoes infected with transgenic *Plasmodium falciparum* (NF54) gametocytes	Transmission inhibition	(34)
6C6	NTPase isozymes	*T. gondii*	*in vitro*: co-colture *Toxoplsma gondii* and Vero Cells	Inhibition of tachyzoites invasion of Vero cells	(35)

**Table 2:** mAb evaluated in clinical trials for protozoan diseases treatment.

**mAb**	**Disease**	**Molecular target**	**Type of ab**	**Trail phase**	**Study aim**	Trial status^*^	**Identifier**
SCH708980	Visceral leishmaniasis	human IL-10	Humanized monoclonal antibody	Phase 1	To study the safety and effectiveness of SCH708980, alone and combined with AmBisome(Registered Trademark), as a treatment for visceral leishmaniasis	Withdrawn (Drug Product no longer available)	NCT01437020
VRC-MALMAB0100-00-AB (CIS43LS)	Malaria—*P. falciparum*	PfCSP—*P. falciparum* circumsporozoite protein	Human monoclonal antibody	Phase 2	To evaluate the safety, tolerability, and efficacy of VRC MALMAB0100-00-AB (CIS43LS) against naturally occurring Plasmodium falciparum (Pf) infection	Recruiting	NCT04329104
VRC-MALMAB0100-00-AB (CIS43LS)	Malaria—*P. falciparum*	PfCSP—*P. falciparum* circumsporozoite protein	Human monoclonal antibody	Phase 1	To evaluate safety and tolerability of different dosages of VRC MALMAB0100-00-AB (CIS43LS) in healthy malaria-naive individuals, as well as the protection against *P. falciparum* following Controlled Human Malaria Infections (CHMI)	Recruiting	NCT04206332
TB31F	Malaria—*P. falciparum*	Pfs48/45, gametocyte surface protein	Humanized monoclonal antibody	Phase 1	To assess the safety and tolerability of mAb TB31F administered intravenously or subcutaneously in healthy, malaria naïve, adults	Completed, results not yet available	NCT04238689

* *Info as per July 17th, 2021*.

*Data retrieved from ClinicalTrials.gov*.

## Leishmaniasis

The NTD leishmaniasis is endemic in 98 countries in the tropics, subtropics, and southern Europe areas (36), with more than 1 billion people being at risk of infection. Twelve million individuals are currently estimated to be infected and every year 2 million new cases are reported, of which 30,000 of visceral leishmaniasis (VL) and more than 1 million of cutaneous leishmaniasis (CL) (37–40). These data are however underestimated as case reporting is mandatory in only 32 countries (41). Leishmaniasis is caused by *Leishmania* spp., an obligate intracellular protozoan that specifically infects macrophages. More than 20 species of *Leishmania* are pathogenic for humans and are transmitted by the bite of a female sandfly. Depending on the species, the parasite can migrate to different organs: to the oropharyngeal cavity-causing mucocutaneous leishmaniasis (MCL), to the viscera causing VL also called Kala-Azar, or it can remain at the skin level causing CL. Leishmaniasis represents a serious public health problem particularly associated with poverty and is responsible for a significant socio-economic burden. VL is the most severe form of the disease since it is lethal if untreated. MCL is a very painful and disfiguring pathology, while CL can cause life-long scars and serious disability or stigma (38–40, 42).

The treatment of leishmaniasis depends on several factors including parasite species, type of disease, co-morbidities, and geographic location (40). Currently available drugs present, however, important drawbacks. Pentavalent antimonials (Sb^V^), meglumine antimoniate (Glucantime®), and sodium stibogluconate (Pentostam®), have been the first-line treatment for leishmaniasis for over 100 years but they have been dismissed for the appearance of resistance and toxicity (43–49). The second-line drugs are pentamidine and amphotericin-B, which however are not optimal for their acute toxicity, high costs, and complex administration requiring hospitalization. Hopes were placed in Miltefosine, a third-line drug against *Leishmania* spp., for which resistance was however detected after only a decade of use (44, 46, 48, 49). Finally, Paromomycin, despite its low cost, requires parenteral administration and resistance has been reported as well (48, 49). Additional information about leishmaniasis treatment is available in the WHO technical report series 949, “Control of leishmaniasis” (50).

Leishmaniasis cure requires an immunocompetent system because available treatments are not able to eliminate the parasite from the body (40). Thus, drugs able to modulate the host immune response might be useful and, over the past years, attempts to find mAb therapies have been made, although they were mostly limited to *in vitro* experiments or animal models. An example is the mAb targeting PD-1 (programmed cell death protein 1), which has been approved by the EMA and FDA for different cancer types (51, 52). PD-1 expressed on T cells mediates macrophages differentiation into classically (M1) or alternatively (M2) activated, through its binding to PD-L1 receptor expressed on macrophages surface (53). When associated with its ligand PD-L1, PD-1 induces the deactivation or apoptosis of antigen-specific T lymphocytes, leading to the suppression of tumor necrosis factor α (TNF-α) secretion (17). Despite the role of the axis PD-1/PD-L1during *Leishmania* spp. infection is still unclear (53), it is known that the differentiation into M2 generates a favorable environment for amastigote survival while the differentiation into M1 leads to parasite death due to the activation of inducible nitric oxide synthase (iNOS) (54, 55).

Although based on pieces of evidence collected through preclinical experimental studies, anti-PD-1/PD-L1 mAbs have been suggested as a potential treatment for leishmaniasis (56). Indeed, PD-1 blocking through mAbs was shown, *in vitro*, to eliminate the intracellular parasites from phagocytes (18) or to reduce the parasite burden *via* the increase of NO, IL-4 and TNF-α in mononuclear cells (17), both isolated from naturally infected dogs. Recently, the first *in vivo* experiment performed in BALB/c mice infected subcutaneously with *L. amazonensis* showed a reduction in the parasite load in mice treated with anti-PD-1 and anti-PD-L1 mAb compared to untreated animals, while the development of cutaneous lesions seemed not to be affected by the biological treatment (56). The therapeutic effects of anti-PD1 or anti-PD-L1 mAb were also suggested to be potentiated by their combination with traditional drugs (56), although this hypothesis has yet to be confirmed.

Anti-CD2 mAb is another promising example of mAb for the control of VL. CD2 is an immuno-modulator and co-stimulatory molecule that induces the endogenous release of IFN-γ by Th1 cells. The combination of anti-CD2 mAbs with the conventional antimonial chemotherapy resulted in the elimination or the control of parasite replication in BALB/c mice inoculated with sensitive or drug-resistant *L. donovani* strains (19).

It has previously been demonstrated that TNF-α is necessary for the control of *Leishmania* spp. infection in a BALB/c mouse model (57). In CL and MCL patients, high levels of TNF-α correlated with major skin lesions (58), while asymptomatic subjects presented moderate levels of TNF-α and INF-γ (59). Following these observations, it was suggested to use anti-TNF-α agents in combination with standard therapy in patients with massive skin sores caused by CL (58). In 2018, Schwartz et al. (60) topically applied anti-TNF-α in combination with paromomycin on CL skin lesions in BALB/c mice infected with *L. major*. The combination therapy led to smaller size lesions while anti-TNF-α monotherapy did not affect lesion size.

mAb against junctional adhesion molecule C (JAM-C)—involved in leukocyte migration through the endothelium—were reported to increase the Th1 response leading to reduced skin lesions and parasite burden in resistant C57BL/6 mice, while it boosted the Th2 response promoting infection in susceptible BALB/c mice (20). These results agree with previous pieces of evidence on the inhibition of the protective Th1 response in resistant mouse strains following the administration of neutralizing antibodies against pro-inflammatory cytokines, such as IL-12 or IFN-γ, and the induction of resistance to *L. major* in susceptible mice following inhibition of Th2 cytokines (21). Anti-IL-4 antibody therapy in BALB/c mice was shown effective against chronic borderline leishmaniasis, leading to lesion reduction and a shift toward a Th1 immune response with anti-parasitic effects (61). A mAb against the regulatory cytokine transforming growth factor β (TGF-β) has been studied in intermediate susceptible CB6F1 mice infected with *L. major* and was shown to induce a rapid lesion healing and a reduction in the number of parasites present in the skin lesion (21).

While promising, to the best of our knowledge these studies have not gone further and the potential use of immunomodulatory mAbs to elicit a Th1 response in the human host has yet to be explored.

Despite several *in vitro* and *in vivo* studies, there is currently only one registered clinical trial (ClinicalTrials.gov Identifier: NCT01437020) (62) for the assessment of the safety and efficacy of anti-IL-10 mAb in combination with amphotericin-B for the treatment of visceral leishmaniasis. Unfortunately, the study was withdrawn due to the unavailability of the drug and no additional information is available.

## Chagas Disease

Chagas disease (CD), or American Trypanosomiasis, is an NTD caused by *Trypanosoma cruzi* and widespread in several countries of Central and South America and in part of Mexico. About 7 million people are estimated to be affected by CD worldwide (63, 64). *T. cruzi* is transmitted by an invertebrate triatomine bug although outbreaks of orally-transmitted infections *via* the consumption of contaminated food have also been reported. Congenital transmission can also occur, while blood transfusions or organ transplants are less common transmission routes. After an initial acute stage with mild and unspecific symptoms, lasting for about 2 months, CD remains silent for several years and becomes chronic. In this stage, the protozoa reach the target organs and in 10–40% of infected subjects can generate cardiomyopathy or mega viscera such as hepatomegaly or splenomegaly (63–65). Despite the efforts to discover novel and safe drugs, anti-*T. cruzi* chemotherapy for both newborns and adults still relies on nifurtimox (NFX) and benznidazole (BZN), two drugs discovered in the '60s with known adverse side effects (64, 66). To improve CD treatment, strategies based on combinations of existing drugs or re-dosing regimens are currently being evaluated (67). Even though the excellent efficacy in reducing the parasitaemia when administered during the acute phase, CD treatment is less effective in preventing the clinical progression when given long time after the initial infection, suggesting a role for the host immune response (68). The mechanisms of interaction between *T. cruzi*, an obligate intracellular protozoa, and the host's immune system are however complex and far from being completely understood, and how the parasite resists, escapes or subverts the host's immune response to establish a chronic infection is still unclear (69).

As for *Leishmania* spp. first-line host defenses against *T. cruzi* depend on the Th1 or Th2 macrophage activation (70). Several studies suggest that the progression to the chronic stage, or its aggravation, might be associated with an immune dysregulation characterized by significantly increased tissue-infiltrating Th1 cells, raised IFN-γ/IL-10 ratio and diminished Treg (71, 72). In children successfully treated with BNZ or NFX it was observed a decline in pro-inflammatory cytokines and chemokines and in T cells expressing IFN-γ and IL-2 along with an increase in IL-7-expressing T cells (73).

The use of mAb to modulate the host immune response might thus have a huge impact on the treatment of chronic CD, although only a few preliminary studies using the murine model are available in the literature.

CD25^+^ Treg, characterized by the production of IL-10 and TGF-β, were reported to be involved in *T. cruzi* infection control, even though their specific role has yet to be completely defined (74). The depletion of CD25^+^ T cells in experimentally infected BALB/c mice using an anti-CD25 mAb, led to a slight increase in IFN-γ and TNF-α production in CD acute stage but not in the chronic stage (22). A different mAb against CD25 was shown to reduce parasitemia and to increase effector memory T cells and IFN-γ/TNF-α-secreting cells in mice when administered during the acute phase, while when administered at the beginning of the chronic phase it was shown to reduce the local inflammatory process in the heart. These results indicate a potential for anti-CD25 mAbs for the treatment of chronic CD and pave the way for more in depth investigations (23). A humanized anti-CD25 mAb, daclizumab, was widely used in the past for the treatment of multiple sclerosis but was withdrawn in 2018 by the EMA because of severe side effects (75). A chimeric anti-CD25 mAb, basiliximab, is currently employed to prevent transplant rejections (76, 77). The use of mAbs against CD25 in mice indicated that Treg inactivation during a *T. cruzi* infection can reduce the number of inflammatory cells, supporting the possibility of using non-depleting monoclonal antibodies as treatment of chronic CD in humans (22, 23).

## Malaria

The most recent WHO estimates reported 229 million malaria cases worldwide in 2019 and 409,000 deaths, about two thirds of which affecting children under the age of 5 (78). Although both malaria incidence and mortality have declined over the past 20 years, the burden associated with this disease remains high. Five Plasmodium species (*P. falciparum, P. vivax, P. malariae, P. ovale*, and *P. knowlesi*) are responsible for human infections and are transmitted by infected female Anopheles mosquitoes. Not surprisingly the majority of the therapeutic and preventive efforts tackle *P. falciparum* since it is responsible for most malaria cases, even though important issues of drug resistance have developed over the years.

Current recommendations foresee the use of artemisinin combination therapies (ACT) as first-line treatment for uncomplicated malaria infections and artesunate for severe malaria, although other less effective drugs such as quinine, chloroquine or other monotherapies are still available and employed in some circumstances (79). This treatment strategy has substantially contributed in reducing malaria morbidity and mortality over the past 15 years, even though resistance development remains a crucial problem threatening malaria control in both South-East Asia and in the African continent (79, 80).

The introduction of effective anti-malaria vaccines has thus become a priority in the fight against this parasite and numerous studies are ongoing to identify novel candidate antigens since the only vaccine currently available, RTS,S/AS01, has limited efficacy (81). In this context, mAbs are also being evaluated for the prevention or treatment of malaria. Compared to a classical vaccination strategy, mAbs have a relatively short-term effect, since IgG have a plasma half-life of approximately 20–25 days (82); consequently anti-parasitic mAbs might be found particularly useful as a preventive measure among high risk populations of severe and potentially fatal complications. Provided that they are proven to lack off-target reactions that could lead to improper immune activation or infectivity enhancement, mAbs against malaria might be used in children, pregnant women, patients with HIV/AIDS, and migrants or travelers with no previous exposure (83).

The first hints on the potential utility of antibodies to fight human malaria infection date back to the '60s when the efficacy of the passive transfer of immunoglobulin from hyper-immune sera to infected individuals was first assessed (84). Different aspects of *Plasmodium* spp. life cycle and malaria pathogenesis need however to be taken into account when considering mAbs as therapeutics or prophylaxis. First of all the mechanisms of host immunity to malaria are yet to be fully deciphered, especially concerning humoral immunity (85, 86). Acquired immunity develops after multiple exposures to the pathogen over the years and, although it mitigates the clinical aspects of the disease, it does not prevent re-infection. This might at least in part explain why, in the African continent, children under the age of 5 are more vulnerable and susceptible to develop clinical and severe forms, which in some cases can be fatal.

The second important aspect to take into account is *P. falciparum* complex life cycle, involving a vector stage and, within the human host, a liver-stage followed by asexual intra-erythrocytic replication (87). Consequently, different mAbs could potentially be designed in order to prevent (i) the infection, if targeting pre-erythrocytic stage (sporozoite); (ii) the clinical disease if targeting asexual erythrocytic stage; (iii) the transmission, if targeting sexual stage (gametocytes) that will not be able to replicate within the mosquito gut (88, 89). Different antigens and epitopes could thus be selected for mAb development, including proteins expressed by merozoites, on the surface of parasitized red blood cells (RBCs) or even by gametocytes. For the successful development of potent mAbs, it is thus essential to better understand the molecular basis of malaria pathogenesis, including erythrocyte invasion mechanisms as well as host immunity, in order to identify highly conserved antigens.

Circumsporozoite protein (CSP), is by far the most investigated target of mAbs for malaria. CSP is the most abundant antigen expressed on sporozoite surface and is involved in parasite attachment to and invasion of hepatocytes through its binding to heparin sulfate proteoglycans (90). The first mAbs against *P. falciparum* and *P. vivax* CSP were developed in the '80s and were shown to reduce parasite infectivity *in vitro* and *in vivo* (91, 92). Since then, a lot of research has focused on anti-CSP antibodies in order to highlight those with the highest affinity and best efficacy in preventing hepatocyte invasion, and to assess their protective properties (24, 25, 88). Thanks to the clinical trials for RTS,S vaccine, which is based on different portions of CSP, mAbs specific to CSP have become available relatively easily and this explains why mAbs anti-CSP are largely studied. Among anti-PfCSP mAbs, CIS43LS is currently undergoing a phase 1 (ClinicalTrials.gov Identifier: NCT04206332) (62) and a phase 2 clinical trial (ClinicalTrials.gov Identifier: NCT04329104) (62) to determine its safety, tolerability, and efficacy.

mAbs against other parasite stages, especially merozoites, have also been evaluated, although their investigation was more in the context of identifying novel vaccine candidates rather than on their direct use as therapeutics. These include mAbs against the merozoite reticulocyte-binding protein homolog 5 (PfRH5), for which a first vaccine has already been developed (26, 27), cysteine-rich protective antigen (PfCyRPA) (28), PfEBA175 (29, 30), apical membrane antigen 1 (PfAMA1) (31), merozoite surface proteins (MSP) (32, 33), all evaluated for their ability to block erythrocyte invasion being all these proteins involved in the interaction between merozoite and RBC.

The intra-erythrocytic stage can also be inhibited by targeting RBC antigens, as it occurs with meplazumab, a humanized anti-CD147 mAb that inhibits RBC invasion by blocking CD147 interaction with rhoptry-associated protein 2 (RAP2) (93). Preclinical studies have shown good tolerability and efficacy of this mAb which, thanks to its mode of action, could be used for both therapy and prophylaxis and will be further evaluated in a phase 1 clinical study (ClinicalTrials.gov Identifier: NCT04327310) (62). Targeting gametocytes specifically has also been proposed, since transmission-blocking antibodies are able to prevent *Plasmodium* spp. transmission from the human host to the vector. TB31F is a humanized version of the rat-derived mAb 85RF45.1 which targets Pfs48/45, a gametocyte surface protein involved in male gamete fertility (34). Indeed, antibodies against Pfs18/45 were reported to inhibit zygote development and thus parasite development within the mosquito (94). A phase 1 clinical trial has recently been completed, evaluating TB31F safety and tolerability in malaria naïve subjects. To the best of our knowledge, however, the results are yet to be published (ClinicalTrials.gov Identifier: NCT04238689) (62).

## Toxoplasmosis

*Toxoplasma gondii* is a single-cell obligate intracellular parasite responsible for human toxoplasmosis. Felids are the definitive host of *T*. *gondii*, in which it completes its life cycle by sexual reproduction, while other warm-blooded animals, including humans, can become intermediate hosts (95–98). The most common infection route for humans is direct contact with cat feces or contaminated soil/water. Food-borne infection can however also occur, especially through the consumption of undercooked meat or contaminated raw fruits and vegetables not properly washed.

*T. gondii* is among the top 10 food-borne pathogens. One-third of the global population is considered to be infected with *T. gondii* (99), but the highest prevalence is found in South America (96, 99, 100).

In immunocompetent subjects, human toxoplasmosis is usually asymptomatic (96, 98, 101, 102), while in immuno-compromised individuals it can manifest as an acute, subacute or chronic disease. Clinical presentation can vary from flu-like symptoms to severe extensive lesions in vital organs such as lungs, liver, heart, brain or eyes (103). The infection can be particularly problematic when acquired during pregnancy as *T. gondii* can pass the placenta and infect the fetus resulting in preterm birth, eye and brain damages including visual and hearing loss, hydrocephaly and microcephaly, or even in fetal death (96–98, 101, 104). During the chronic stage, the parasite enters into a “sleeping” form, called bradyzoite, that forms cysts that colonize different tissues preferentially brain, muscles and eyes (96, 98, 103, 105). Chronic toxoplasmosis is resistant to anti-parasitic therapies and consequently can undergo reactivation/recrudescence (101).

Immune competent subjects recover spontaneously from asymptomatic *T. gondii* infection, while a combination of antimicrobial agents such as pyrimethamine and sulfadiazine can be administered to symptomatic individuals during the acute phase (98, 106). These drugs present very toxic side effects and cannot be used for a prolonged time. As indicated by the CDC, pregnant women, newborns, infants and immunocompromised patients can require discontinuation of therapy and, as a consequence, parasites will not be completely eliminated (98, 107). During the first decade of 2000, a mAb against nucleoside triphosphate hydrolase (NTPase) was investigated. NTPases are unique enzymes produced by *T. gondii* and released into the parasitophorous vacuoles (35); they are responsible for the hydrolysis of ATP to ADP and ADP to AMP and their activity leads to *T. gondii* tachyzoite intracellular survival and replication (108). NTPase activity was shown to be proportional to tachyzoite multiplication rate (109) and mAbs against NTPases were reported to inhibit *in vitro* the enzymatic activity in a dose-dependent manner in different *T. gondii* strains, limiting *T. gondii* invasion and replication within host cells (35, 110). The same enzyme was also evaluated as a candidate antigen for the development of a toxoplasmosis vaccine. Indeed, a “suicidal” DNA vaccine, later upgraded in self-amplifying RNA linked to lipid nanoparticle, based on NTPase-II sequence, was synthesized and shown, in mice, to elicit a Th1 immune response associated with a higher percentage of CD8^+^ T cells, increased levels of INF-γ, IL-2 and IL-10, and decreased IL-4. This vaccine partially reduced the rate of infection and mortality in both acute and chronic infections, however in-depth studies are yet to be performed (111, 112).

A number of different toxoplasma antigens have been proposed in the last years as candidates for the development of monoclonal antibodies or vaccines, although only preliminary studies have been performed so far. These include rhoptry kinase 18 of *Toxoplasma gondii* (TgROP18), a key virulence factor that promotes parasite proliferation for which species- and strain-specific mAbs have been obtained (113), and a synthetic peptide from surface antigen 1 (SAG1) (114).

## Other Protozoan Diseases

### *Trichomonas vaginalis* and *Cryptosporidium* spp.

To the best of our knowledge, preliminary experimental studies for the use of mAbs as therapeutics have also been performed for human trichomoniasis and human cryptosporidiosis, although the subject has not been addressed in depth. In the case of *Trichomonas vaginalis*, an extracellular parasite, it was demonstrated that the inhibition of parasite adhesion to epithelial cells through mAbs reduces parasite motility and protects from infection both *in vitro* and in the mouse model (115–117). The use of bovine hyper-immune colostrum or mAb against different *Cryptosporidium* spp. antigens, an apicomplexan protozoon like *Plasmodium* spp., was instead proposed in the '80s and the '90s to reduce disease severity in human cryptosporidiosis as summarized by Mead (118), although investigations were not pursued further.

### *Trypanosoma brucei* spp.

A particular case is represented by *Trypanosoma brucei spp*., the causing agent of human African trypanosomiasis (HAT), for which classical mAbs have not been investigated. This extracellular protozoa has in fact evolved important mechanisms in order to survive in the human bloodstream, involving both immune escape and immune-suppression (119, 120), consequently, antibody-mediated responses were shown to have scarce efficacy in parasite elimination. This also explains why all attempts were undertaken so far to identify an anti-HAT vaccine have failed (121, 122). Nonetheless, an interesting alternative to classical mAb is represented by nanobodies (Nbs), small engineered antibody fragments derived from the heavy chain-only antibodies of camelids, characterized by high stability, specificity for their target, and tolerability (123). Their potential for therapeutic applications for HAT has been recently reviewed by Stijlemans et al. (124) and Nbs against variant surface glycoprotein (VSG) epitopes were reported to display trypanolytic effects *in vitro* and *in vivo* (125), supporting the potential of this novel technology.

## Considerations and Conclusions: The Expert Point of View

During the COVID-19 pandemic, the anti-IL-6 mAb Tocilizumab (TCZ) has emerged as an effective treatment (126). Subsequently, 47D11, a human mAb that binds to cells expressing the viral spike protein, was shown to neutralize SARS-CoV-2 *in vitro* (127). At present, we count more than hundred clinical trials for SARS-CoV-2 treatment (62), for which a promising strategy to at least limit the spread of the virus could be represented by the use of cocktails of neutralizing antibodies (128). The great efforts put in place to fight this pandemic have led to rapid and effective results. Unfortunately, only a few clinical studies are currently ongoing to evaluate the use of mAbs for the treatment or prophylaxis for parasitic infections (Table 2). A number of reasons could explain the paucity of these clinical studies or the failure of many candidates at the pre-clinical level. First, most protozoan diseases are NTDs, affecting the poorest populations in low income countries. mAbs, mainly employed for the treatment of disorders affecting industrialized countries, especially cancers and auto-immune diseases, had an estimated market of about 98 billion USD in sales in 2018 (129), which is expected to increase of about 20% per year, reaching 138.6 billion USD in 2024 (129, 130). Although their use is now well-diffused, the costs of production are still very high and so is the price for the final customer, i.e., the patient or the health care system (131). Consequently, at the current state, it is not economically conceivable to employ such an expensive therapeutic strategy for the fight against NTDs. The case of malaria, which is not listed among NTDs, confirms this first point. More financial efforts have been applied to fight this disease (132, 133) and as a result is the only protozoan disease having several clinical trials ongoing for therapeutic use of mAbs.

Another important factor that could explain the difficulties associated with the introduction of mAb for protozoan diseases, is the complexity of the mechanisms of host-pathogen interaction. Despite great efforts to improve our knowledge of parasite biology and of the mechanisms of parasitism, many aspects are yet to be fully deciphered. Consequently, the translation of evidence collected through *in vitro* and *in vivo* experimental studies is often hardly translatable to the more complex human condition. Indeed, experimental mouse models can mimic only few aspects of human pathologies and in some cases different parasite strains need to be employed as human-infecting strains might not be pathogenic in mice (134–136). Another important consideration that needs to be done in the context of parasitic diseases, is the indirect effect that mAb employed for the treatment of other disorders might have on host susceptibility to infections or on the reactivation of latent ones as observed, for instance, for latent leishmaniasis (137) or latent Chagas disease (138).

In the future, one possibility would be to increase the scale production and the stability of the mAb products, in order to considerably decrease the costs (83). A promising alternative is represented by nanobodies, as previously mentioned for HAT. Besides their lower costs and smaller size, these tools have the advantage of being more stable, which make them attractive for disease prophylaxis. Their potential has been investigated not only for HAT; for instance, a single-chain fragment variable (scFv) against *T. gondii* SAG1, able to achieve a more rapid distribution and better tissue penetration compared to the whole antibody, was reported to efficiently reduce the number of oocyst and the mortality burden in a murine model of congenital toxoplasmosis (139). An engineered single-chain variable fragment derived from mAb-10D8 (scFv-10D8), was shown to adhere to *T. cruzi* surface and to reduce infectivity in pre-treated cultures, suggesting a potential use of such antibodies as preventive biological drugs (140).

Novel technologies and bioinformatics approaches should thus be exploited to achieve a more in depth knowledge of parasite biology and identify novel and highly specific targets to *ad-hoc* design mAbs. Such a strategy was in fact already found useful for malaria, for which a number of clinical studies evaluating anti-CSP mAb are currently ongoing.

Strategies based on drug repurposing, in order to employ mAb able to modulate host-immunity to limit damages due to the infection and hyper-inflammation, have also failed. Parasites are in fact well-known to develop effective mechanisms to escape host immune response. Nonetheless, a successful approach might consist in the combination of mAbs with classical anti-parasitic drugs as already proposed for leishmaniasis (19, 60).

In conclusion, mAbs are yet to be found useful in clinical practice for the treatment or prevention of protozoan infections. The main obstacles to the development of mAbs or Nbs therapies to parasitic diseases appear to be related to the production costs and the complexity of the host-pathogen interaction. Considering the rapidity of technology improvement in the development of biologicals, we can foresee that in the near future there would be the possibility for the application of these therapies also for parasitic infections, provided that the preclinical and clinical research can better define the host-parasite mechanisms and reveal the key targets for a specific mAbs or Nbs treatment.

## Author Contributions

SL: conceptualized the manuscript. SL and NT drafted the manuscript. SL, NT, ZB, and CP reviewed and edited the manuscript. All authors have read and agreed to the submitted version of the manuscript.

## Funding

This work was supported by the Italian Ministry of Health Fondi Ricerca Corrente—L3P2 to IRCCS Sacro Cuore Don Calabria Hospital.

## Conflict of Interest

The authors declare that the research was conducted in the absence of any commercial or financial relationships that could be construed as a potential conflict of interest.

## Publisher's Note

All claims expressed in this article are solely those of the authors and do not necessarily represent those of their affiliated organizations, or those of the publisher, the editors and the reviewers. Any product that may be evaluated in this article, or claim that may be made by its manufacturer, is not guaranteed or endorsed by the publisher.
